# Single-cell RNA sequencing of platelets: challenges and potential

**DOI:** 10.1007/s11239-025-03153-8

**Published:** 2025-07-31

**Authors:** Giacomo Viggiani, Kilian Kirmes, Jiaying Han, Melissa Klug, Stephanie Kühne, Gianluigi Condorelli, Karl-Ludwig Laugwitz, Conor J. Bloxham, Clelia Peano, Philip Raake, Isabell Bernlochner, Dario Bongiovanni

**Affiliations:** 1 Department of Internal Medicine I, University Hospital Rechts Der Isar, Technical University of Munich, Munich, Germany; 2 German Center for Cardiovascular Research (DZHK), Partner Site Munich Heart Alliance, Munich, Germany; 3 Department of Internal Medicine I, University Hospital Augsburg, University Augsburg, Augsburg, Germany; 4 Department of Cardiovascular Medicine, Humanitas Clinical and Research Center IRCCS and Humanitas University, Rozzano, Milan, Italy; 5 Institute of Genetics and Biomedical Research, UoS of Milan, National Research Council, Milan, Italy; 6 Human Technopole, Via Rita Levi Montalcini 1, Milan, Italy

**Keywords:** Platelets, Single-cell RNA sequencing, Thrombocyte biology, 10X genomics, Platelet rich plasma

## Abstract

**Abstract:**

Platelets are small, anuclear cells crucial for hemostasis, coagulation, immune responses, and vascular diseases. While unable to produce their own RNA, platelets inherit RNA from their megakaryocyte precursors, exchange RNA with other cells, and possess all the necessary machinery for protein synthesis. However, several challenges, including their limited RNA content, high reactivity of these small cells leading to their activation, have hindered single-cell transcriptomic studies of these cells. The primary objective of this study is to perform single-cell RNA sequencing (scRNA-seq) on platelets obtained from whole blood. Peripheral whole blood from a healthy donor was obtained by venipuncture and was purified to obtain platelet-rich plasma (PRP). ScRNA-seq was performed using the 10X genomics platform on PRP for the first time. Data normalization and UMAP clustering with cluster-specific differential gene expression analysis were performed. ScRNA-seq performed on platelets identified three distinct clusters, with one enriched for platelet-specific lineage markers, such as *PPBP* and *PF4*. Mitochondrial RNA was highly expressed accounting for approx. 14% of the total RNA counts. Despite procedural challenges and technical considerations including high exhaustion potential and sensitivity to handling, small cell size and limited RNA content, this pilot study demonstrates feasibility of scRNA-seq of platelets from whole blood. This advancement paves the way for groundbreaking insights into platelet biology and more focus for clinician researchers on potential research avenues.

**Graphical abstract:**

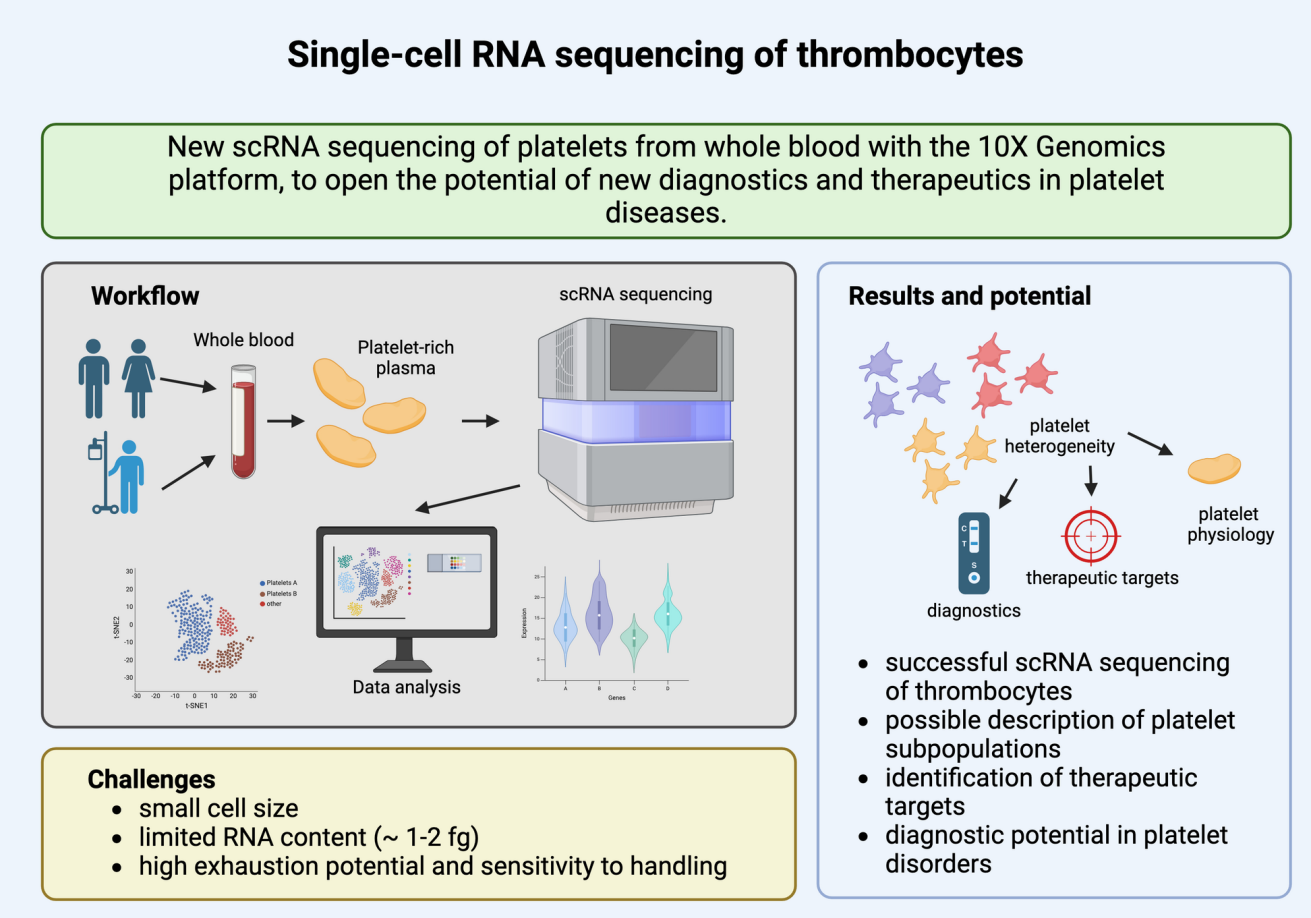

## Highlights

- Single-cell RNA sequencing (scRNA-seq) is a promising yet underutilized technique for platelet biology.
- This pilot study successfully performed scRNA-seq on platelets from whole blood, identifying three distinct clusters, with one enriched for platelet-specific markers.
- Despite inherent challenges, this innovative approach highlights the potential to uncover platelet subpopulations associated with thrombotic risk and therapeutic outcomes.
- scRNA-seq could drive advances in cardiovascular and hematologic diagnostics, enable therapy stratification, and advance personalized medicine.
- By establishing a foundation for future research, this study paves the way for deeper insights into platelet heterogeneity and its clinical relevance.

Platelets are small, anucleated cells that play a central role in thrombosis, hemostasis, and immune responses, making them critical in cardiovascular and hematologic conditions. Platelet inhibition remains a cornerstone of therapy for many cardiovascular diseases. However, the transcriptomic heterogeneity of platelets and their subpopulations—arising from selective mRNA transfer from megakaryocytes during thrombopoiesis and RNA decay during maturation—remain poorly understood [1, 2]. This heterogeneity may underpin differences in platelet aggregation potential and functional roles, with significant implications for diagnostics and therapeutic strategies.

Emerging evidence highlights the existence of platelet subpopulations, such as reticulated platelets, which are immature, hyper-reactive, and highly thrombogenic. These cells are elevated in coronary syndromes and in high risk cohorts [3–5], underscoring their clinical relevance. Despite this scRNA-seq, a powerful tool for investigating cellular heterogeneity, has been sparsely applied to platelets, with only a few anecdotal reports [6, 7].

The unique biological characteristics of platelets—including their small size, minimal RNA content (1–2 femtograms per cell), and sensitivity to activation—pose significant technical challenges for single-cell isolation and transcriptomic analysis. However, overcoming these hurdles could provide transformative insights into platelet biology, revealing novel subpopulations and their roles in disease and treatment response.

In this study, we conducted exploratory scRNA-seq of platelets isolated from whole blood from a healthy donor using the 10X genomics platform for the first time. Peripheral blood was collected via venipuncture into citrate vials and processed to obtain platelet-rich plasma (PRP), as described before [8]. Sample preparation was optimized to avoid high-shear procedures such as filtration or sorting. Minimal centrifugation was applied to preserve quiescence. Timing and temperature control were strictly maintained during sample handling to minimize ex vivo activation. Approximately 50,000 cells were loaded onto the 10X platform for scRNA-seq [9, 10].

**Fig. 1:**
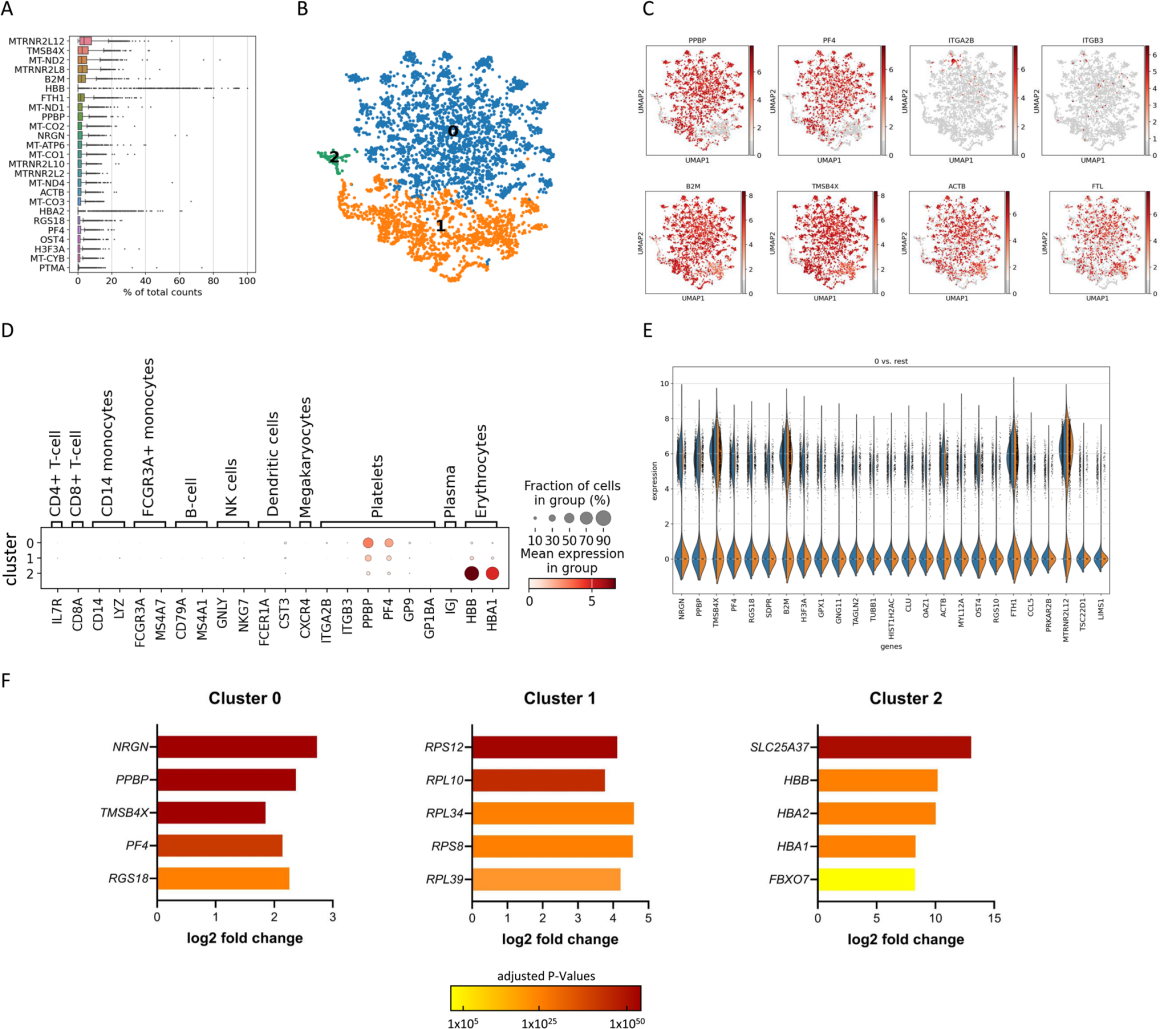
Single-cell sequencing of platelets isolated from a healthy donor. **A** scRNAseq revealed mitochondrial gene transcripts and platelet-related gene transcripts among the 25 most expressed genes. **B** Low resolution clustering analysis identified 3 distinct clusters of cell phenotypes, characterized by the 10 most highly differentially expressed genes based on RNA expression. **C** Cluster distribution of single gene transcripts showed that *PPBP* and *PF4* are predominant in cluster 0. **D** Lineage marker expression per cluster indicated that the majority of cells in the sample were platelets. **E** Comparative gene expression of the 25 highest expressed genes in cluster 0 (blue) versus pooled clusters 1 and 2 (orange) confirmed the specificity of platelet markers in cluster 0. **F** Clusterwise ranking of highly differential genes in platelets. The top differentially regulated genes for each of the three clusters are presented, including the gene name, log2 fold change, and adjusted p-value (using the Benjamini–Hochberg procedure). scRNAseq: single-cell RNA sequencing

The analytical pipeline followed Seurat’s clustering methodology outlined by Satija et al*.* [11], with adaptations to accommodate the unique characteristics of platelets. The scripts for bioinformatic analysis can be found in the supplementary material in the aforementioned work of Satija et al.. *Scanpy*, a scalable toolkit for analyzing single-cell gene data, was used for the analysis and the rendering (https://scanpy.readthedocs.io/en/stable/). To maximize data capture, no filtering thresholds were applied to avoid unintended exclusion of RNA-poor, mature platelet subpopulation data, which may otherwise be lost due to low transcript complexity, andmitochondrial RNA, known to be abundant in platelets, was retained [12, 13]. Data normalization and UMAP clustering were performed, with cluster-specific differential gene expression analysis conducted using the Wilcoxon rank-sum test. Clustering analysis was projected as described before using the Benjamini–Hochberg procedure for false discovery rate (FDR) control [14]. Comparative analysis focused on the 25 highest expressed genes in cluster 0 relative to the other clusters. In total, 4092 cells were successfully sequenced, yielding 32,738 detectable genes. The 25 most highly expressed transcripts were ranked (Fig. 1A). A significant proportion of the sequencing reads corresponded to thrombocyte-specific RNA (Fig. 1A–C). Consistent with findings from previous platelet RNA-seq studies [12, 13], mitochondrial RNA was highly expressed accounting for 14.4 ± 0.2% of the total RNA counts (Fig. 1A), with transcripts such as *MTRNR2L12* and *MT-ND2*, among the most abundant [12, 13]. Specific platelet RNA transcripts including *PPBP*, *OST4* and *PF4*, were also among the top-expressed genes, corroborating an earlier study [15]. Low-resolution clustering identified 3 major clusters (0–2), characterized by the 10 most highly differentially expressed genes within each cluster (Fig. 1B; Fig. 1F). Differential genes in the clusters are shown in Fig. 1C. Cluster 0, the largest, exhibited the highest expression of platelet markers (Fig. 1C–F), confirming the predominance of platelets in the dataset. Cluster 1 was enriched with ribosomal transcripts, while cluster 2 displayed transcripts associated with erythroid lineage (*SLC25A37*, *HBB*, *HBA1* and *HBA2*) [15], suggesting minor erythrocyte contamination (only ~ 1.4% of the detected cells) (Fig. 1F). A cell lineage marker analysis (Fig. 1D), further highlighted that cluster 0 had the strongest expression of platelet-specific markers A comparison of the 25 most highly expressed genes in cluster 0 with the pooled clusters 1 and 2 reaffirmed the enrichment of platelet specific markers in cluster 0 (Fig. 1F).

Here we present one of the first scRNA sequencing studies of platelets from a healthy donor. Despite its exploratory nature, our findings confirm the feasibility of scRNA-seq for platelets. However, the resolution of our current analysis was insufficient to identify distinct sub-populations. Future advancements in methodology could enhance resolution and provide further insight into platelet heterogeneity and functional diversity.

This study highlights the potential of scRNA-seq as a tool to investigate platelet heterogeneity, paving the way for advancements in both clinical research and practice. Key translational applications could include: (1) identifying platelet subpopulations linked to thrombotic risk, therapeutic resistance (e.g., P2Y12 inhibitor low response [16–19]), or bleeding disorders; (2) stratifying patients at higher risk of thrombotic events based on platelet transcriptomic profiles; (3) improving our understanding of hematologic conditions, such as essential thrombocythemia, myeloproliferative disorders, and immune thrombocytopenia, where platelet diversity influences disease mechanisms or treatment outcomes [20–23]. Future work should prioritize refining isolation techniques to minimize sample contamination and developing robust clinical workflows to incorporate scRNA-seq findings into diagnostics and personalized therapeutic strategies.

Our pilot analysis has several limitations, primarily stemming from the low RNA input. To preserve as much data as possible, no filtering was applied for genes per cell or cells per gene. While this approach aimed to maximize the analyzable dataset, it inherently complicated the identification of pure platelet (sub-)populations and introduced potential biases. Additionally, the presence of platelet aggregates in the system cannot be ruled out. Future studies might address this by pooling platelets from different species (e.g., mice and humans), to estimate aggregate burden, which warrants further investigation.

Another limitation arises from the reliance on PRP to avoid platelet activation. Although effective in reducing activation, this approach sacrifices sample purity. Magnetic bead-based depletion or sorting platelets, using CD41 as a marker, could enhance sample purity but may expose platelets to high shear stress, potentially inducing activation or aggregation. We deliberately chose not to apply these techniques in this pilot study to minimize mechanical and biochemical stress that may induce platelet activation and confound transcriptomic profiles. Exploring alternative low-shear sorting strategies could offer a viable solution for future studies.

With a number of successfully sequenced cells less than 10% of the total number of cells loaded into the 10X platform, it may be possible that RNA-poor platelets are missed by this scRNA-seq technology. Given the very low RNA content of MPs (1–2 femtograms per cell), it is likely that a substantial proportion of cells failed to yield sufficient RNA for capture and library preparation, in our study. This inefficiency may result from platelet heterogeneity and RNA content among platelets, platform limitations (e.g., dropout events, poor cell-bead pairing with small cells), or losses during filtering steps like barcode assignment and cell calling in *Cell Ranger*. These processes may exclude low-complexity libraries generated from RNA-poor cells. While the exact contribution of each factor is unclear, current scRNA-seq platforms may systematically underrepresent mature platelets.

scRNA-seq experiments with different cell types achieve better results, probably due to the higher content of RNA and the size of the cell. Furthermore, it has been well documented that young reticulated platelets (RPs) are RNA-rich compared to mature platelets (MPs), who display a loss of 20–40% RNA content. This emphasizes the distinct molecular profile of RPs compared to MPs [24]. A different distribution of RNA content in platelets introduces a bias that should be considered when interpreting results. However, while RPs are important, particularly in clinical cardiovascular scenarios, we would like to stress that sequencing of MPs, which represent the majority of circulating platelets, is not only feasible but also provides valuable insights into platelet biology. Previous transcriptomic studies by our group demonstrated the ability to sequence MPs and analyze their transcriptome, even in the context of their reduced RNA content [2]. However, to date, no single surface marker robustly distinguishes RPs from MPs at the single-cell level. As a result, a stratification of this sort would likely require prior sorting based on RNA content or age-specific markers, followed by scRNA-seq. Future studies designed specifically for RP vs. MP analysis, ideally integrating surface phenotype and transcriptomic data, would be valuable for delineating their distinct molecular profiles and clinical implications.

In addition, the lack of detection of a specific population could be due to overrepresentation of RNA-rich reticulated platelets in this RNA concentration sensitive approach, as mentioned before. From a technical point of view, a possible improvement could be offered by other technologies, such as nanowell and visually confirmed single cell capture systems [25], which use a large-bore nozzle dispenser for unbiased cell size isolation, allowing the inclusion of the smaller and RNA-poor mature platelets.

In the future, scRNA-seq should also be performed on activated platelets to possibly determine subpopulations of thrombocytes that would not be recognized otherwise.

Nevertheless, platelet activation carries intrinsic technical difficulties including exhaustion and particularly aggregation, which would make it infeasible with the used 10X genomics platform. Other approaches for scRNA-seq, for example the aforementioned nanowell and visually confirmed single cell capture systems, could overcome this limitation and allow such as interesting analysis. Furthermore, systematic evaluation of activation-related transcriptomic changes, ideally via comparison to bulk sequencing data from stimulated platelets, could provide important insights into platform-induced activation. This approach should be a priority for future investigations aimed at benchmarking technical artifacts in platelet scRNA-seq.

ScRNA sequencing has long been underutilized in platelet research due to inherent challenges such as platelets’ small size, minimal RNA content, and high reactivity. Nevertheless, in this study, we successfully performed exploratory scRNA sequencing on platelets from a healthy donor, as evidenced by the abundance of platelet-specific RNA reads. This preliminary investigation demonstrates the potential of scRNA-seq to explore platelet heterogeneity, though further refinements in techniques and platforms are essential to unlock its full utility in platelet biology research.

## Data Availability

Data is provided upon reasonable request.
